# Catalytic Nanoceria Are Preferentially Retained in the Rat Retina and Are Not Cytotoxic after Intravitreal Injection

**DOI:** 10.1371/journal.pone.0058431

**Published:** 2013-03-11

**Authors:** Lily L. Wong, Suzanne M. Hirst, Quentin N. Pye, Christopher M. Reilly, Sudipta Seal, James F. McGinnis

**Affiliations:** 1 Department of Ophthalmology, University of Oklahoma Health Sciences Center, College of Medicine, and Dean McGee Eye Institute, Oklahoma City, Oklahoma, United States of America; 2 Biomedical Sciences and Pathobiology, Virginia Polytechnic Institute and State University, and Via College of Osteopathic Medicine, Blacksburg, Virginia, United States of America; 3 Advanced Materials Processing Analysis Center, Mechanical Materials Aerospace Engineering, Nanoscience and Technology Center, University of Central Florida, Orlando, Florida, United States of America; 4 Department of Cell Biology and Oklahoma Center for Neuroscience, University of Oklahoma Health Sciences Center, Graduate College, Oklahoma City, Oklahoma, United States of America; University of Sydney, Australia

## Abstract

Cerium oxide nanoparticles (nanoceria) possess catalytic and regenerative radical scavenging activities. The ability of nanoceria to maintain cellular redox balance makes them ideal candidates for treatment of retinal diseases whose development is tightly associated with oxidative damage. We have demonstrated that our stable water-dispersed nanoceria delay photoreceptor cell degeneration in rodent models and prevent pathological retinal neovascularization in *vldlr* mutant mice. The objectives of the current study were to determine the temporal and spatial distributions of nanoceria after a single intravitreal injection, and to determine if nanoceria had any toxic effects in healthy rat retinas. Using inductively-coupled plasma mass spectrometry (ICP-MS), we discovered that nanoceria were rapidly taken up by the retina and were preferentially retained in this tissue even after 120 days. We also did not observe any acute or long-term negative effects of nanoceria on retinal function or cytoarchitecture even after this long-term exposure. Because nanoceria are effective at low dosages, nontoxic and are retained in the retina for extended periods, we conclude that nanoceria are promising ophthalmic therapeutics for treating retinal diseases known to involve oxidative stress in their pathogeneses.

## Introduction

Nanomaterials which include nano-sized and nano-structured objects, have gained importance in biomedical research and medicine in recent years. Because of the dramatic increase in surface area when synthesized in the nanometer range, nanomaterials exhibit enhanced or unique reactivity that is not found in their macroscopic counterparts. Many promising nanomaterials are currently under investigation for drug or nucleic acid delivery to target specific organ/tissue for therapy. Others are tested for diagnostic, imaging, tissue healing, and surgical aids [1]. Another unique class of nanomaterials, namely the redox-active radical scavenging nanoparticles including fullerenes and cerium oxide nanoparticles (nanoceria or CeNPs), is being developed as bona fide antioxidants for treatment of neurodegenerative diseases [2]–[4].

The oxides of cerium, a rare earth element, have unique physical and chemical properties. The cerium ions have both the 3+ and 4+ valence states and therefore can act as electron donors or acceptors. Oxygen defects or vacancies on the surface or subsurface of the lattice crystals act as sites for radical scavenging [5], [6]. When synthesized in the 3–5 nm range, nanoceria possess enhanced catalytic activities that mimic superoxide dismutase and catalase [7]–[9], two major anti-oxidative enzymes, to neutralize superoxide anions and hydrogen peroxides, respectively. The enhanced redox capacity of nanoceria is most likely due to the dramatically increased surface to volume ratio of these nanoparticles.

Accumulating evidence has shown that the disease progression of many neurodegenerative conditions such as Alzheimer’s, Parkinson’s and retinal degenerative diseases including age-related macular degeneration, diabetic retinopathy, and various forms of retinitis pigmentosa, are tightly associated with oxidative damage due to either chronically or acutely increased reactive oxygen species [10]–[16]. During the past few years, we have focused on developing our stable water-dispersed nanoceria as ophthalmic therapeutics for treatment of retinal diseases. We showed that these nanoceria increased the lifespan of retinal neurons in culture and protected them from oxidative damage when challenged with hydrogen peroxide [17]. Nanoceria synthesized using the same methodology also protected photoreceptor cells in a light-induced retinal degeneration model [17]. They inhibited the development and caused the regression of pathologic retinal neovascularization in the *very low density lipoprotein receptor* (*vldlr*) knockout mouse [18]. These nanoceria also delayed the degeneration of photoreceptor cells in a retinal degeneration mouse carrying the *tubby* mutation [19].

Despite the well-documented ability of nanoceria to reduce oxidative damage, retinal degeneration, and inflammation [17]–[20], the mechanisms of radical scavenging by nanoceria in biological systems are still unclear [2], [21]. Furthermore, the bio-distribution and pharmacokinetics of nanoceria in ocular tissues after a single intravitreal injection (the optimal route for nanoceria delivery) are unknown. Nanoceria appear to have differential effects in different cell types. From cell culture studies, certain cell types exhibited enhanced longevity and protection from oxidative insults while a few showed reduced viability when exposed to nanoceria at specific dosages [2], [22]. Currently, a systematic study of nanoceria cytotoxicity *in vivo* in ocular tissues is lacking. We therefore carried out a detailed study to specifically address these fundamental questions to characterize the interactions of nanoceria in the unique biological environment of the eye. We used inductively-coupled plasma mass spectrometry (ICP-MS), a highly sensitive method for trace element detection in biological samples, to study the bio-distribution of nanoceria after a single intravitreal injection in the rat eye. We discovered that nanoceria were rapidly and preferentially retained in the retina for at least 120 days. We also showed that nanoceria were not toxic to retinal cells over a range of dosages applied. Our study is the first to demonstrate that nanoceria are retained in the retina for an extended period after a single intravitreal injection and that nanoceria do not have toxic side effects in retinal cells *in vivo* at the dosage levels applied.

## Materials and Methods

### 1. Animal

We kept a breeding colony of Sprague-Dawley (SD) albino rats in the Dean McGee Eye Institute (DMEI) vivarium under cyclic light conditions (12 h on/12 h off, 5–20 lux).

### 2. Ethics Statement

Animals were cared for and handled according to the Association for Research in Vision and Ophthalmology statement for the use of animals in vision and ophthalmic research. The study was approved by the University of Oklahoma Health Sciences Center Institutional Animal Care and Use Committee (OUHSC IACUC) and the DMEI IACUC. The approved protocol numbers were 10-087 and 10-088 from the OUHSC IACUC, and D-10-087 and D-10-088 from the DMEI IACUC.

### 3. Synthesis of Nanoceria

Cerium oxide nanoparticles were synthesized using simple wet chemistry methods as described previously [23]. Briefly, stoichiometric amounts of cerium nitrate hexahydrate (99.999% from Sigma Aldrich) were dissolved in deionized water. The solution was oxidized using an excess amount of hydrogen peroxide. After the synthesis of nanoparticles, the pH of the suspension was maintained below 3.0 using nitric acid (1M) to keep the synthesized nanoceria in suspension. These 3–5 nm particles were thoroughly characterized using transmission electron microscopy (size and shape determination), dynamic light scattering (zeta potential measurement), and X-ray photoelectron spectroscopy (estimating the oxidation state of cerium) as described in [18]. Each batch was validated for the abundance of catalytically active Ce3+ oxidation state and stable aqueous dispersion. More importantly, we did not use hexamethylenetetramine (HMT) in the synthesis of nanoceria due to cytotoxic effects exhibited by nanoparticles prepared in this manner (unpublished observation).

### 4. Intravitreal Injection of Nanoceria

Adult SD rats (8 weeks or older) were selected for intravitreal injection. Animals were anesthetized by intramuscular injection of a mixture of ketamine (80 mg/kg) and xylazine (4 mg/kg). Pupils were dilated by application of a drop of phenylephrine (10% solution) to the cornea before the delivery of 2 µl of either CeNPs of varying dosages (1 µM or 0.344 ng to 1 mM or 344 ng in saline solution) or saline alone into the vitreous with the aid of an ophthalmic operating microscope. Both eyes of each animal received the same treatment.

### 5. Sample Collection for Inductively Coupled Plasma Mass Spectrometry (ICP-MS)

Eyes were harvested at designated times post injection. Enucleated eyes were fixed in 4% (para-formaldehyde) PFA (in 0.1M phosphate buffer, pH 7.4) at 4°C until ICP-MS analysis. If further dissection was performed, eyes were fixed at room temperature for 30 minutes before dissection. Eyes were dissected into component parts in cold PBS, pH 7.4. Ocular components: retina (R), lens (L), rest of eyecup including cornea, iris, retinal pigment epithelium, choroid, and sclera (EC) or whole eyes were kept in individual Eppendorf tubes containing 1 ml 4% PFA and stored at 4°C until processing for ICP-MS. In most cases (80%), the vitreous body was found associated with the lens tissue and was included in the lens component. When the vitreous body was not associated with the lens tissue, it was discarded in the dissecting buffer. We confirmed that inclusion of the vitreous body in the lens component did not alter the amount of nanoceria in the lens component.

### 6. ICP-MS

Tissues in 1 ml fixative were mixed in 10 ml 70% nitric acid overnight to start the digestion process. Samples were then microwave-digested in an Xpress Microwave Digester. The temperature was ramped to 200°C over a span of 20 minutes and held there for another 20 minutes. Samples were then boiled down to less than 1 ml each and reconstituted in water to 10 ml exactly. The Ce levels were assessed using a 7700 Series ICP-MS from Agilent Technologies (Santa Clara, CA). The level of Ce was converted to CeO_2_ for data presentation. Each time point represented the average from at least four eyes from two individual rats.

### 7. Electroretinogram Recordings (ERG)

Animals were dark adapted overnight and manipulated under dim red light. Anesthesia was induced with a mixture of ketamine (80 mg/kg i.m.) and xylazine (4 mg/kg i.m.) and the animal was placed on a thermal water heated pad with the temperature controlled (38°C) by a circulating water pump (GAYMAR T/PUMP, Orchard Park, NY). Pupils were dilated at least five minutes before testing using a topical phenylephrine (10%) solution (AK-DILATE, Akorn, Inc., Lake Forest, IL). A drop of 2.5% hypromellose solution (GPS, Wilson Ophthalmic, Mustang, OK) was applied to the cornea to prevent dehydration and allow for electrical contact with the gold recording electrode (Goldring Electrode, 4 mm, Roland Consult, Stasche & Finger GmbH, Brandenburg, Germany). A platinum subdermal needle (Grass Technologies, West Warwick, RI) hooked into the mouth, right side of the cheek, served as the reference electrode. To complete the circuit, a platinum subdermal needle electrode was placed under the skin at the base of the tail. The Espion system from Diagnosys LLC (Lowell, MA) provided amplification (at 0.30 to 300 Hz bandpass, with notch filtering), stimulus presentation, and data acquisition.

#### Scotopic

The scotopic stimuli consisted of white flashes provided by a xenon bulb projected on a ganzfeld. The intensity of stimuli evaluated and specifics of the protocol are shown in Table 1. The amplitude of each A-wave response corresponded to the maximum negative deflection found between 6 and 35 ms after the stimulus. The amplitude of each B-wave response corresponded to the difference between the maximum negative deflection determined and the maximum positive deflection found between 30 and 130 ms after the stimulus.

**Table 1:** Parameters of the nine steps used for Scotopic ERG recording.

Step	Light intensity (cd.s/m^2^)	Light intensity Logscale (cd.s/m^2^)	# trials per result	Inter trial delay (s)
1	0.006	−2.22184875	4	10
2	0.009	−2.045757491	4	10
3	0.03	−1.522878745	3	15
4	0.06	−1.22184875	3	15
5	0.3	−0.522878745	3	20
6	3	0.477121255	3	25
7	30	1.477121255	3	30
8	300	2.477121255	2	50
9	600	2.77815125	2	60

#### Photopic

The source of photopic stimuli was the same as the one presented for scotopic stimuli and flashes were presented consecutively at 0.06, 0.6, 3.0, 30.0, 300.0 and 600.0 cd.s/m2. Prior to the first flash, a five minute light adaptation sequence was used at a luminance of 30 cd/m^2^. A total of 10 responses were averaged for each intensity tested with an inter trial delay of 1 s. We determined the amplitudes of each A-, and B-wave response as described under the Scotopic condition. Only B-wave amplitudes are presented.

#### Flicker

The flicker stimuli at 30 cd.s/m^2^ flash intensity with a background luminance of 30 cd/m^2^ were presented at 3 Hz, 5 Hz and then at 10–40 Hz in 10 Hz steps. For each flicker frequency tested, the stimulus was presented followed by a 5 s delay prior to data collection. This guaranteed that the first few responses (not preceded by repeated stimuli and of potentially greater amplitude) were excluded. The wave trains were examined to ensure consistency of responses over the duration of stimuli (500 ms). Amplitude from the first wave form from each frequency was determined. Finally, presentation of the 3 Hz stimulus was repeated to ascertain the stability of the responsiveness, i.e. confirming that amplitudes obtained with this last trial were comparable to those obtained with the first trial. The amplitude of each flicker response corresponded to the difference between the first maximum negative and the first positive deflections. These protocols were modified from the ones described in [24], [25]. Measurements from these ERG recordings enable us to evaluate the functions of several classes of retinal neurons. Scotopic a-wave amplitude reflects primarily the function of rod cells. Scotopic b-wave amplitude reflects the function of neurons in the inner retina, post-synaptic to the rod photoreceptor cells. Photopic b-wave amplitude and flicker ERG reflect the function of cone cells [26]. Each data point represented the average from at least three individual rats or six eyes.

### 8. Histology and Morphometric Analysis

**Figure 1:**
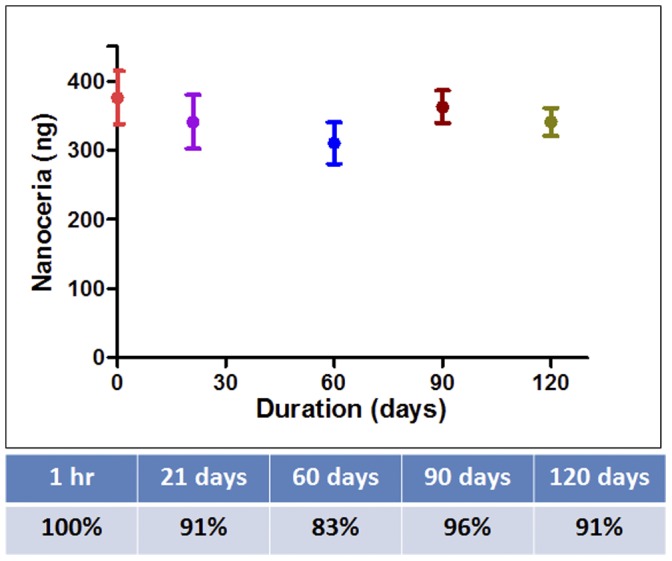
Parameters for evaluation of effects of nanoceria on retinal cytoarchitecture. **A.** Schematic diagram of a cross section of a rodent eye cut through the optic nerve head. The thick black line in the posterior part of the globe represents the retina. Measurements were taken from i) the central portion of the retina: 960 µm from the optic nerve head (ONH), and ii) the peripheral portion of the retina: 960 µm from the ora serrata, along the superior and inferior axis of the globe. Each marked interval represents ∼960 µm. **B.** Photomicrograph of an H&E stained retinal section to illustrate the measurements taken from different retinal layers for quantitative analyses. Inner retina is up. 1 = retina thickness (RT) = inner limiting membrane (ILM) to outer limiting membrane (OLM), 2 = inner nuclear layer (INL), 3 = outer nuclear layer (ONL), 4 = inner and outer segments (IOS) of rod cells. To minimize the effects of uneven tissue shrinkage among eye samples, we normalized the thickness of INL, ONL, and IOS by comparing these measurements with the overall thickness of the retina (RT). Measurements from a single retinal section were taken using a calibrated reticle on one of the binoculars of a Nikon E400 microscope under a 20X objective. Averages were from eyes of different animals.

After ERG recordings, the rats were euthanized with carbon dioxide, the eyes enucleated and fixed in Prefer fixative (Anatech Ltd., Battle Creek, MI) for 45 min. Five µm thick paraffin sections were obtained along the superior/inferior axis of the globe through the central retina. Hematoxylin and Eosin (H&E) stained sections were used for morphometric analyses. Detailed description of each measurement is provided in Figure 1. Each data point was from 2–5 eyes from different rats; all but 2 data points had samples of at least 3 eyes.

### 9. Statistical Analysis

For ICP-MS data, each data point was averaged from 4–8 eyes whereas ERG data were averaged from 6–10 eyes. Values were expressed as means ± SEM. Statistical analyses were performed using one-way ANOVA followed by the Tukey multiple comparison tests comparing every group with every other group using GraphPad Prism version 5.00 for Windows (GraphPad Software, San Diego CA USA, www.graphpad.com). A P value less than 0.05 was considered significant and is indicated by an asterisk.

## Results

### 1. Characterization of Nanoceria

Each batch of synthesized nanoceria was thoroughly characterized using (1.) transmission electron microscopy for size and shape determination, (2.) dynamic light scattering for zeta potential measurement, and (3.) X-ray photoelectron spectroscopy for determination of the relative abundance of 3+ and 4+ oxidation state of cerium. The detailed characterization results can be found in the supplemental materials in [18]. Our stable water-dispersed nanoceria are 3–5 nm in size. The size of these particles remains the same in a wide range of pH buffers and upon aging [27]. The surface charge on nanoceria is an important consideration for their interactions with the biological environment. We established that the zeta potential of our synthesized nanoceria at 1 mM in saline solution to be +10±3 mV [18].

### 2. Ninety Percent Of Injected Nanoceria Stayed in the Eye for 120 Days

**Figure 2:**
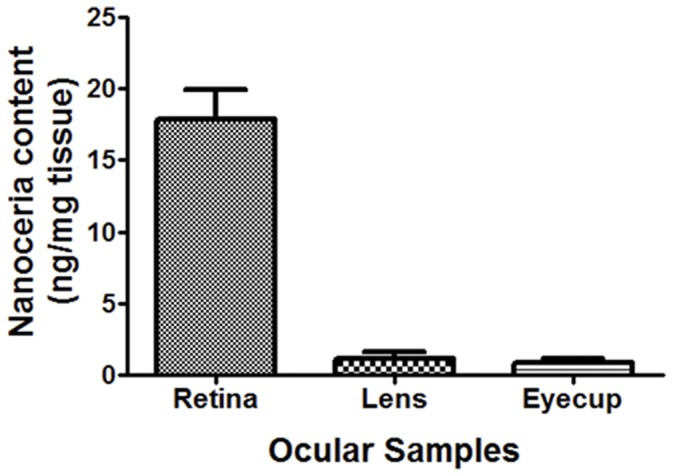
Nanoceria were retained in the eye for months. The amount of nanoceria retained in the eye was determined by ICP-MS. The elimination was extremely slow with approximately 90% of the injected nanoceria still retained in the eye at 120 days or four months post injection.

Since we did not know how quickly nanoceria were cleared in the eye, we injected 1000X the effective dose previously used in our light-damage model in rats [17]. Each eye received 2 µl of 1 mM nanoceria (344 ng). We harvested the eyes at different times after injection: from 1 hour to 120 days. The Ce levels were assessed using a 7700 Series ICP-MS from Agilent technologies that was capable of detecting Ce at a minimum range of 50–100 parts per trillion. **Figure 2** shows the retention of nanoceria in the eye after a single intravitreal injection. This data set shows the combined amount of nanoceria from each eye that had been dissected into component parts: retina, lens, and rest of eye cup. Another experiment, in which whole eyes were analyzed at 1 hour and 30 days post injection, showed similar results (data not shown). Surprisingly, we found that 90% of the injected nanoceria remained in the eye after 120 days. Unlike many ophthalmic agents such as peptides, nucleic acids or organic compounds that are cleared from the eye within hours or days, we observed that nanoceria were not actively eliminated from the eye. Additional retention data from 8- and 12-month time points became available after our submission. We determined that the elimination half-life of nanoceria in the eye and in the retina to be 525 days and 414 days, respectively. These data are presented in **Figures S1 and S2**.

### 3. Nanoceria were Rapidly and Preferentially Taken up by Retinal Cells

**Figure 3:**
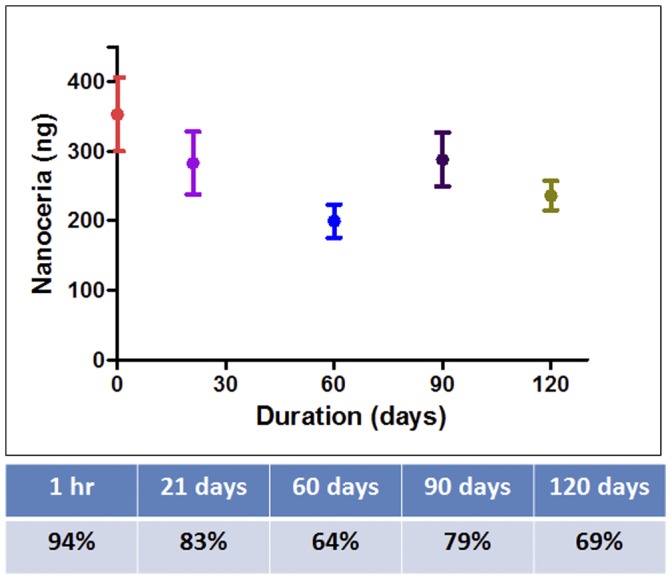
Bio-distribution of nanoceria in ocular tissues one hour post injection. We detected the highest concentration and amount of nanoceria in the retinal portion of the eye whereas the lens and the rest of the eye cup retained only small amounts of nanoceria one hour post injection.

To determine where nanoceria were distributed after injection, we dissected the eye into component parts. We separated the retina and the lens from the rest of the eyecup before ICP-MS analysis. **Figure 3** shows the bio-distribution of nanoceria one hour post injection. During this initial hour, retinal tissue accumulated the highest concentration of nanoceria (17.89 ng/mg tissue, followed by the lens tissue (1.13 ng/mg tissue), and the lowest in the eyecup tissue (0.83 ng/mg tissue). Uninjected eyes contained negligible amount of nanoceria.

**Figure 4:**
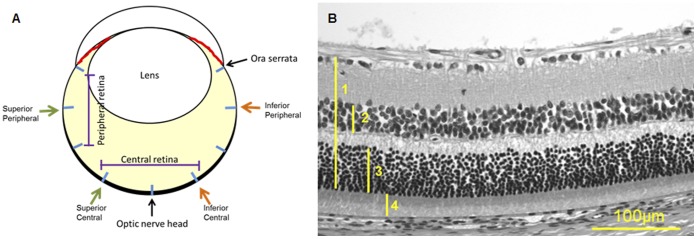
The retina retained nanoceria over prolonged periods of time following a single intravitreal injection. The injected nanoceria accumulated in the retina rapidly. We detected 94% of the injected nanoceria in the retina after one hour of injection and about 70% of the injected nanoceria was retained for the four months tested.

To determine if nanoceria were also preferentially retained in retinal tissue, we analyzed retinal samples from 1 hour to 120 days post injection. **Figure 4** shows the amount of nanoceria remaining in the retina over the 120-day period. We observed that about 70% of the injected nanoceria were retained in the retina even after 120 days. (New data from 8- to 12- month samples can be found in **Figure S2**.).

### 4. Nanoceria did not have Cytotoxic Effects in the Retina

**Figure 5:**
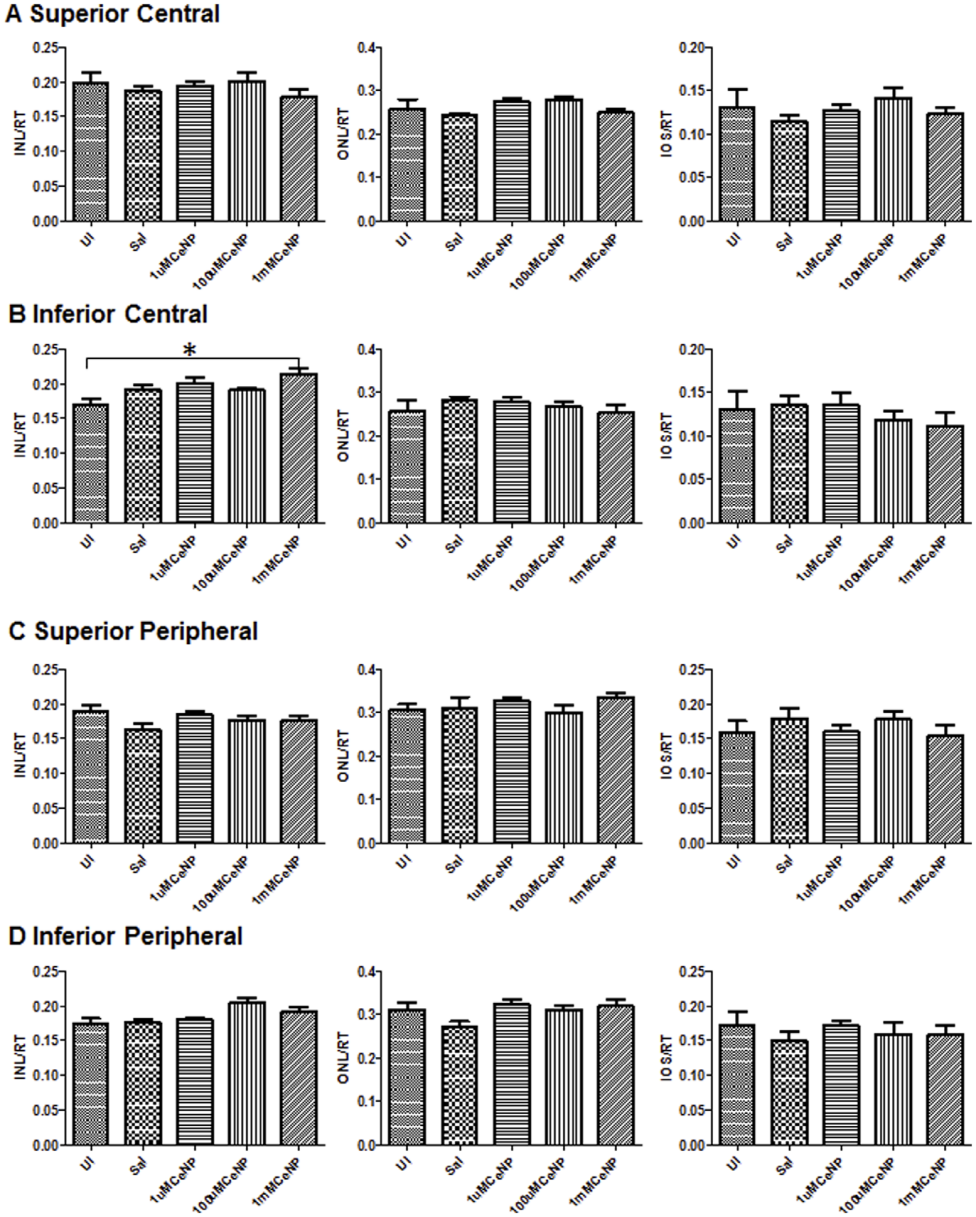
Nanoceria had no toxic effects on the morphological structures of the retina at nine days post injection. We performed quantitative morphometric analyses (see Figure 4 legend) on the central (A–B), and peripheral (C–D) portions of the retina of animals from different treatment groups (UI = uninjected, Sal = saline injected, or CeNP injected with the indicated dosage) and found no changes induced by nanoceria. Representative photomicrographs of retinal sections from this time point are shown in Figure S3.

**Figure 6:**
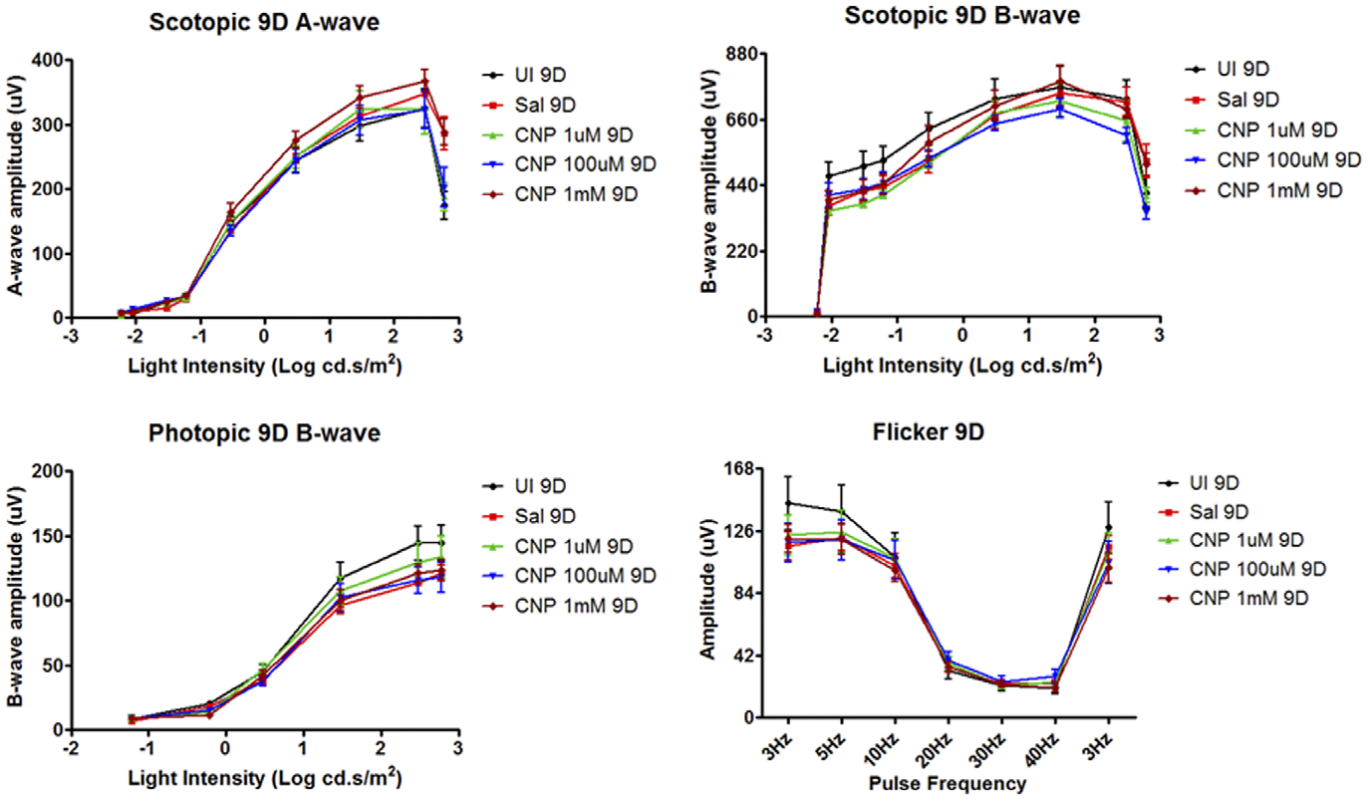
Nanoceria had no negative effects on retinal function as evaluated by ERG recordings nine days post injection. We did not observe changes in amplitudes of scotopic a- and b-waves, photopic b-wave, and flicker at the frequencies indicated.

**Figure 7:**
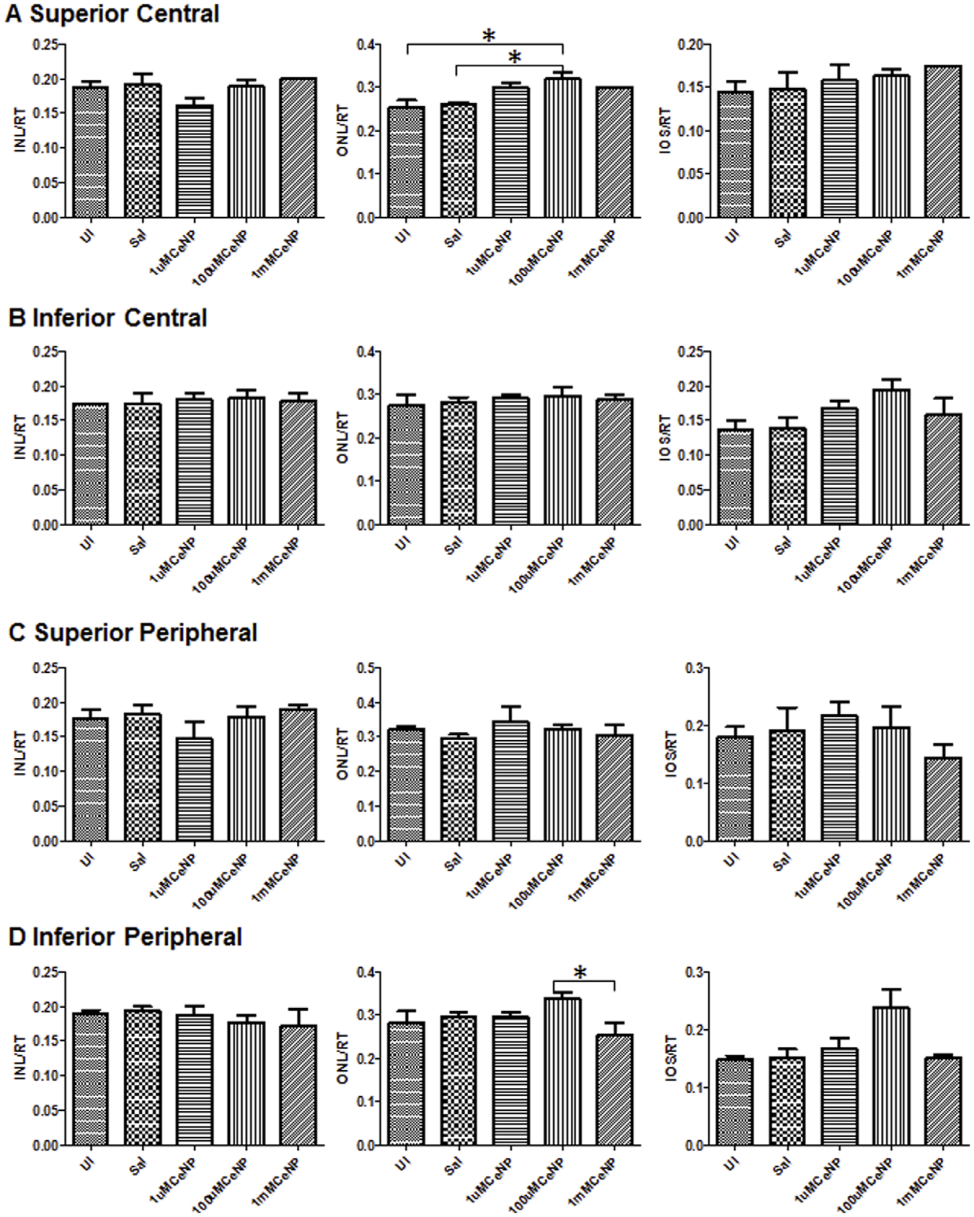
Nanoceria (1–1000 uM) had no negative effects on retinal morphology, even four months after injection, as measured by quantitative morphometric analyses. We examined the central (A–B), and peripheral (C–D) portions of the retina of animals from different treatment groups (UI = uninjected, Sal = saline injected, or CeNP injected with the indicated dosage), and found no changes induced by nanoceria. Measurements were performed as indicated in Figure 4.

To assess the safety of nanoceria for therapeutic treatment, we ascertained the effects of nanoceria in the retina after 9, 60 and 120 days post intravitreal injection. We administered a range of nanoceria dosages (1 µM, 100 µM, and 1 mM) to the rat eyes, and evaluated potential morphological and functional changes in the retina. For retinal cytoarchitecture assessment, we examined the thicknesses of the inner and outer retina (**Figure 1B**). The layer thickness reflects the number of neurons residing in these two layers, thus the overall health of the retina. We also included the thickness of the rod inner and outer segments (IOS) to further evaluate the health status of rod cells. We focus on the rod photoreceptor cells because they are one of the key light sensors and are the most abundant cell type in many mammalian retinas, including rodents and primates [28], [29]. They are also exquisitely susceptible to oxidative damage due to the unusually high content of polyunsaturated fatty acids [30]. Evaluation of the thickness of IOS, therefore, can serve as an indicator of the health status of these cells. To evaluate functional changes, we conducted scotopic, photopic, and flicker full field ERG recordings. Scotopic a-wave amplitude reflects primarily the function of rod cells. Scotopic b-wave amplitude reflects the function of neurons in the inner retina, post-synaptic to the rod photoreceptor cells. Photopic b-wave amplitude and flicker ERG reflect the function of cone cells [26]. **Figure 5** shows the morphometric data from 9 days post injection. From the four surveyed areas: superior and inferior central retina, superior and inferior peripheral retina (see **Figure 1A** for orientation), we did not observe any reduction in thickness in the layers examined for nanoceria injected eyes. **Figure S3** shows representative H&E stained retinal sections from the inferior central portion of each eye for this set of the experiment. We also did not observe any changes in retinal functions among the nanoceria injected versus the saline injected animals (**Figure 6**). These results indicate that nanoceria did not cause acute negative side effects in the healthy retina. Similar results were obtained for the 60 days (data not shown) and the 120 days (**Figures 7** **,** **8** **, and S4**) data sets. From these results, we conclude that nanoceria generated according to our described formulation and procedure, are not toxic to the rat retina as measured by morphology or function, even when 344 ng are present for over 4 months.

**Figure 8:**
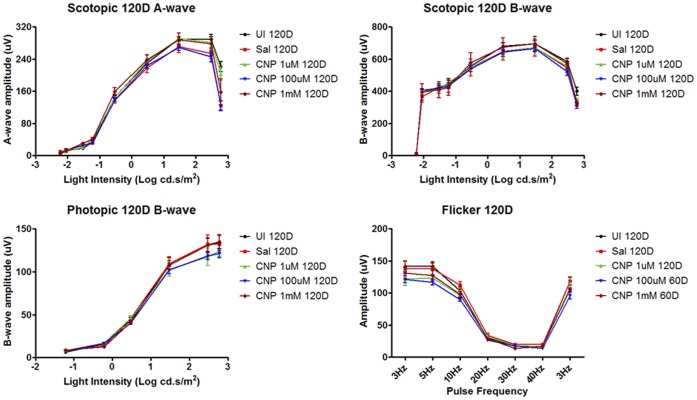
Nanoceria (1–1000 uM) had no negative effects on retinal function, even four months after injection, as measured by ERG recordings. No changes were detected in amplitudes of scotopic a- and b-waves, photopic b-wave, and flicker at the frequencies indicated.

## Discussion

This is the first *in vivo* study to show that nanoceria are rapidly and preferentially taken up and retained in the retina after a single intravitreal injection. The retention of nanoceria in the retina does not have any short- or long-term cytotoxic effects. This study is a more targeted approach to understanding the bio-distribution and side-effects of nanoceria in rodent ocular tissues. The lack of toxic effects in the retina is consistent with our previous findings demonstrating that weekly systemic administration of nanoceria in mice did not have cytotoxic effects in the heart, kidney, brain, lungs, spleen, and liver for a 5-week period [31].

We do not yet know how nanoceria are taken up by retinal cells. However, in an *in vitro* uptake experiment, Singh and colleagues [32] showed that fluorescein-conjugated nanoceria were taken up by keratinocytes via clathrin-, and caveolae-dependent endocytic pathways. These fluorescently-labeled nanoceria were found in multiple cellular compartments including the mitochondria, lysosomes, endoplasmic reticulum, the nucleus, and the cytoplasm. At this moment, kinetic studies on elimination or exocytosis of nanoceria by cells are not available. However, in this *in vivo* study, we established that the elimination half-life in the rat retina to be 414 days (see Supplemental Materials). Our results suggest that the rate of elimination by cells is extremely slow. By 120 days, we observed 30% reduction of injected nanoceria in the retina. Could these nanoceria be accumulating in other organs? We detected trace amounts of nanoceria in liver and kidney tissues from 120 days animals from both nanoceria injected and uninjected animals (data not shown). Our findings suggest that nanoceria are removed from the eye eventually but the primary route of elimination is unknown. It can be by general circulation or locally in the eye, or both. The protracted retention of nanoceria in the eye could be beneficial if the self-regenerative property of nanoceria [33] is maintained. Presently, we have shown that nanoceria are not toxic to retinal neurons after 120 days of exposure and the prolonged retention in the retina should be an asset for the investigation and development of nanoceria as ophthalmic therapeutics.

We are just beginning to unravel the interactions of nanoceria in the complex biological environment. Reports of the differential effects of nanoceria on a variety of cell types in cell culture experiments add to the challenge of developing nanoceria for therapeutic treatments. One major defining feature in these cell culture studies that was seldom discussed was the synthesis and characterization of nanoceria administered [2]. For example, the usage of hexamethylenetetramine (HMT) in the synthesis of nanoceria might contribute to the cytotoxic effects of nanoceria (unpublished observations). The effects of particle aggregation might be another contributor to the negative effects of nanoceria observed in some cell culture studies [34], [35]. The surface charge of nanoparticles plays a major role in their uptake by cells [34]. Asati and colleagues [36] showed that polymer-coated nanoceria with a positive charge were taken up by more cell types than the negatively-charged or neutrally-charged particles. They also demonstrated that the differently charged particles could be localized in the same or different cellular compartments depending on the cell type.

Delivery of nanoparticles to their site of action without dilution poses another challenge. Kim and colleagues [37] compared the movement of fluorescently-labeled human serum albumin nanoparticles with either positive or negative surface charge, in the vitreous and retina of the rat eye. Five hours after intravitreal administration, the authors found that the negatively-charged nanoparticles penetrated the vitreal barrier more easily and were found deep in the retina from the inner limiting membrane to all the nuclear layers and plexiform layers and even in the retinal pigment epithelium. The seemingly contradictory findings from cell culture versus *in vivo* studies with respect to nanomaterial surface charge and uptake illustrate the complexity of the interactions between nanomaterials and biological environments. We caution that the observations made regarding interactions of nanomaterials on synthetic membranes or lipid bilayers and nanomaterials uptake in cell culture studies do not necessarily reflect the actual interactions in biological environments. For *in vivo* systemic delivery, the administered nanomaterials need to circulate in the blood to reach their target tissue. En route, the nanomaterials are likely to have serum proteins adsorbed on the surface through electrostatic or hydrophobic interactions. This adsorption may change the properties of how the nanomaterials will be taken up by target cells.

In this study, we demonstrated that nanoceria administered in the vitreous were able to penetrate through this complex structure and reach the underlying retinal cells. The mammalian vitreous body is a network of collagen fibers embedded in a matrix of highly water-bound glycosaminoglycans [38]. Albumin constitutes about 40% of the soluble proteins in the vitreous [38] and may adsorb to the injected positively-charged nanoceria by electrostatic interactions [39]. However, this potential interaction did not impede the uptake of nanoceria by retinal cells. One unusual feature of the vitreous is the high percentage of iron-binding proteins present compared to the plasma. Van Bockxmeer and coworkers [40] showed that about 35% of the soluble proteins in the monkey vitreous were composed of transferrin and/or lactoferrin. The high transferrin or transferrin-like proteins in the vitreous may contribute to the rapid uptake of nanoceria by retinal cells. We [41] previously showed that nanoceria with positive surface charges can bind to transferrin and were taken up by cells more efficiently than naked nanoceria at a number of concentrations. Transferrin receptors are present in all cell types in the body including retinal cells. We postulate that the rapid uptake of nanoceria may be facilitated by binding to transferrin and/or lactoferrin in the vitreous. This hypothesis is consistent with the observation that nanoceria-mediated photoreceptor cell protection is not focal but is found across the entire retina rather than being confined to the injection site [17].

In spite of the many unknown interactions of nanoceria in the vitreous and other ocular tissues, our results suggest that our stable water-dispersed nanoceria are likely to be taken up by one or more cell types in the retina by an active process. The enhanced redox capacity of nanoceria does not have negative effects in healthy retinal cells. Finally, nanoceria appear to have an extremely slow rate of removal once inside cells.

Currently, effective and comprehensive therapies for blinding diseases such as age-related macular degeneration, diabetic retinopathy, and retinitis pigmentosa are still unavailable. Targeting the reduction of reactive oxygen species by nanoceria in these diseased eyes may be one way to prolong the life and function of retinal cells before cures become a reality. Additionally, drug delivery to the posterior segment of the eye, such as the retina, is most effective when administered intravitreally. This procedure, though simple, carries the risk of complications such as infection and retinal detachment, especially when frequent, repeated applications are required. Taken together, nanoceria appear to be ideal candidates as ophthalmic antioxidants because the redox activity of nanoceria is regenerative [33] and frequent repetitive dosing may be avoided. We predict that nanoceria may become the “aspirin” of the 21st century for the therapeutic treatment of eye diseases whose pathologic progression associates tightly with oxidative damage.

## Supporting Information

Figure S1 **Detection of nanoceria in the eye after one year.**
(PDF)

Figure S2 **Detection of nanoceria in the retina after one year.**
(PDF)

Figure S3 **Photomicrographs of H&E stained retinal sections from adult SD rats nine days post nanoceria (CeNP) intravitreal injection.**
(PDF)

Figure S4 **Photomicrographs of H&E stained retinal sections from adult SD rats 120 days post nanoceria (CeNP) intravitreal injection.**
(PDF)
