# Correction: Catalytic Nanoceria Are Preferentially Retained in the Rat Retina and Are Not Cytotoxic after Intravitreal Injection

**DOI:** 10.1371/annotation/569989ba-586e-468d-bba3-d9a737b15459

**Published:** 2013-09-09

**Authors:** Lily L. Wong, Suzanne M. Hirst, Quentin N. Pye, Christopher M. Reilly, Sudipta Seal, James F. McGinnis

Figures 1 and 4 were incorrectly switched. The image currently appearing as Figure 1 belongs with the title and legend for Figure 4, and the image currently appearing as Figure 4 belongs with the title and legend for Figure 1. 

