# Supplementary material for: Catalytic Nanoceria Are Preferentially Retained in the Rat Retina and Are Not Cytotoxic after Intravitreal Injection
Source: PLoS One. 2013 Mar 11;8(3):e58431. doi: 10.1371/journal.pone.0058431 (PMC3594235; doi:10.1371/journal.pone.0058431)
Supplement: Figure S1 — Detection of nanoceria in the eye after one year. (PDF) [file pone.0058431.s001.pdf]

Retention of nanoceria in the eye after a single intravitreal injection

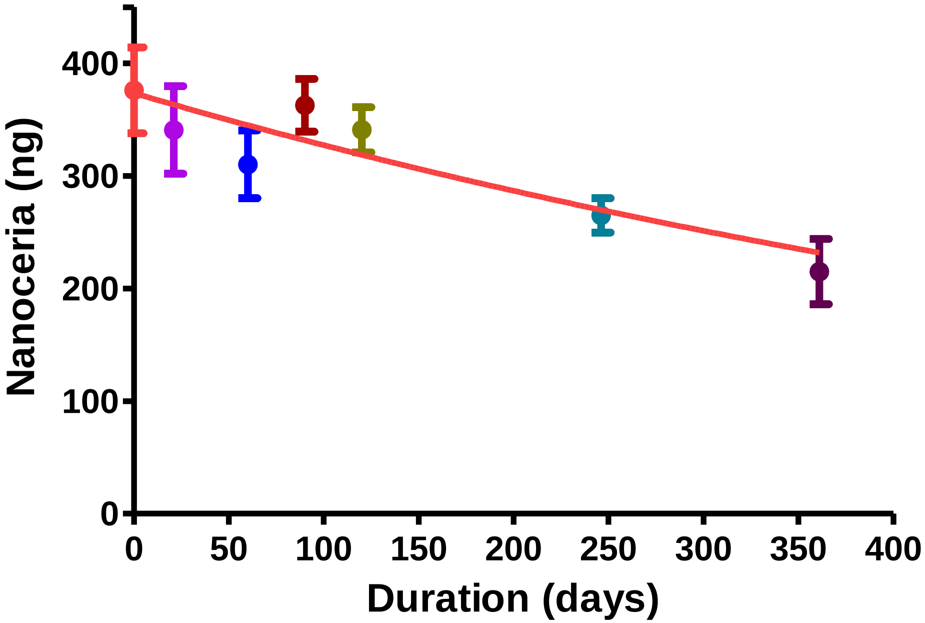

Half life of nanoceria in the eye is 525 days.
