# Supplementary material for: Catalytic Nanoceria Are Preferentially Retained in the Rat Retina and Are Not Cytotoxic after Intravitreal Injection
Source: PLoS One. 2013 Mar 11;8(3):e58431. doi: 10.1371/journal.pone.0058431 (PMC3594235; doi:10.1371/journal.pone.0058431)
Supplement: Figure S4 — Photomicrographs of H&E stained retinal sections from adult SD rats 120 days post nanoceria (CeNP) intravitreal injection. (PDF) [file pone.0058431.s004.pdf]

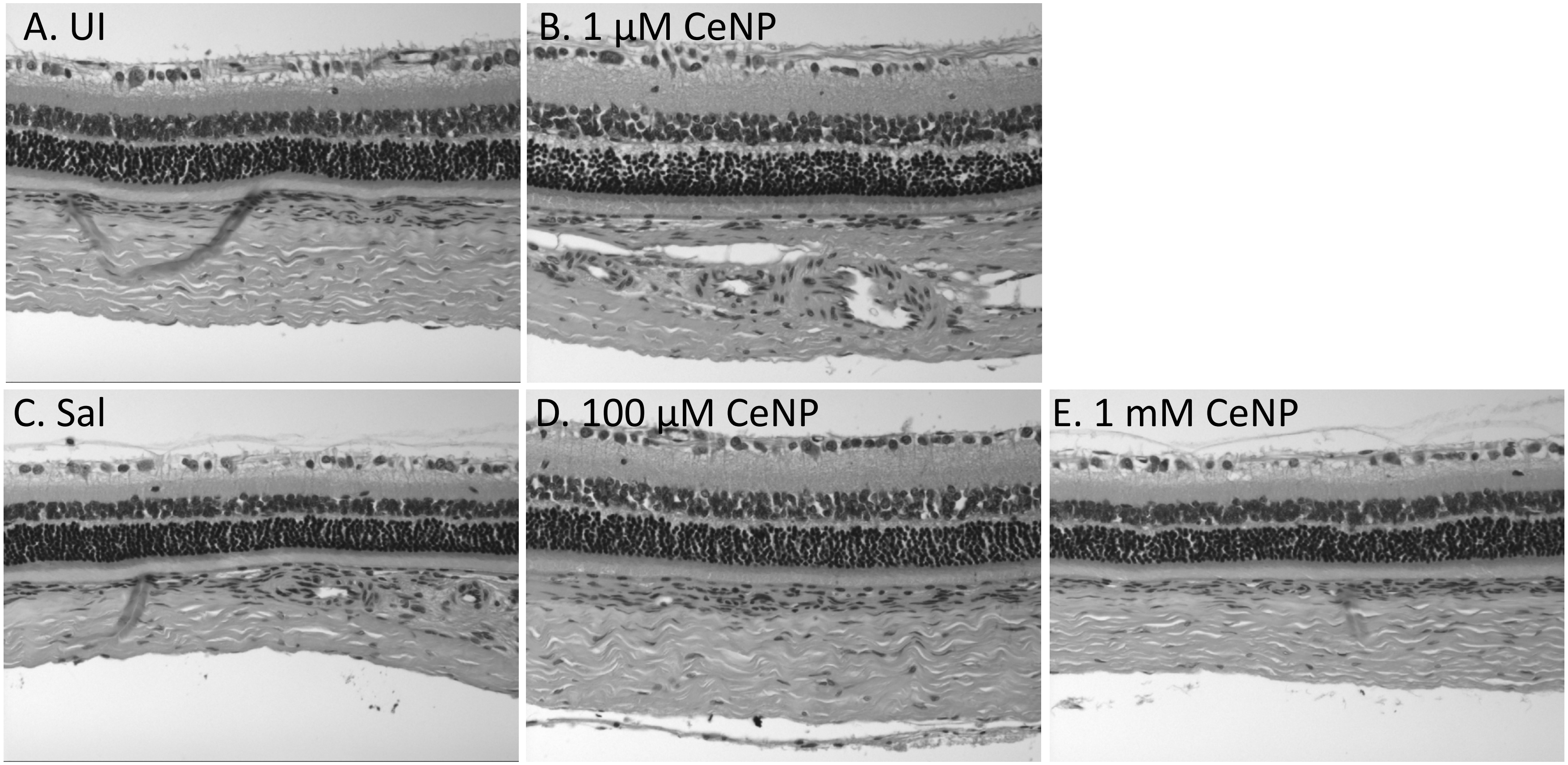

Photomicrographs of H&E stained retinal sections from adult SD rats 120 days post nanoceria (CeNP) intravitreal injection. Images were taken at the same magnification using the 20X objective of a Nikon E800 microscope. We obtained sections through the central retina, i.e. through the optic nerve head. All images were taken at ~1mm from the optic nerve head in the inferior aspect of the retinal section. Abbreviations: UI=uninjected, Sal=saline injected, CeNP=nanoceria injected with the concentration indicated.
